# Myoclonus and Dystonia as Recurrent Presenting Features in Patients with the SCA21-Associated *TMEM240* p.Pro170Leu Variant

**DOI:** 10.5334/tohm.858

**Published:** 2024-04-10

**Authors:** Ugo Sorrentino, Luigi M. Romito, Barbara Garavaglia, Mario Fichera, Isabel Colangelo, Holger Prokisch, Juliane Winkelmann, Jan Necpal, Robert Jech, Michael Zech

**Affiliations:** 1 Clinical Genetics Unit, Department of Women’s and Children’s Health, University of Padova, Padova, Italy; 2 Institute of Neurogenomics, Helmholtz Munich, Neuherberg, Germany; 3 Institute of Human Genetics, Technical University of Munich, School of Medicine, Munich, Germany; 4 Parkinson and Movement Disorders Unit, Department of Clinical Neurosciences, Fondazione IRCCS Istituto Neurologico Carlo Besta, Milan, Italy; 5 Medical Genetics and Neurogenetics Unit, Fondazione IRCCS Istituto Neurologico Carlo Besta, Milan, Italy; 6 DZPG, Deutsches Zentrum für Psychische Gesundheit, Munich, Germany; 7 Munich Cluster for Systems Neurology (SyNergy), Munich, Germany; 8 2nd Department of Neurology, Faculty of Medicine, Comenius University, Bratislava, Slovakia; 9 Department of Neurology, Zvolen Hospital, Zvolen, Slovakia; 10 Department of Neurology and Centre of Clinical Neuroscience, General University Hospital and First Faculty of Medicine, Charles University, Kateřinská30, 12 800, Prague, Czech Republic; 11 Institute for Advanced Study, Technical University of Munich, Garching, Germany

**Keywords:** Pro170Leu, TMEM240, SCA21, Spinocerebellar ataxia, Dystonia, Myoclonus

## Abstract

**Background:**

Spinocerebellar ataxia 21 (SCA21) is a rare neurological disorder caused by heterozygous variants in *TMEM240*. A growing, yet still limited number of reports suggested that hyperkinetic movements should be considered a defining component of the disease.

**Case Series:**

We describe two newly identified families harboring the recurrent pathogenic *TMEM240* p.Pro170Leu variant. Both index patients and the mother of the first proband developed movement disorders, manifesting as myoclonic dystonia and action-induced dystonia without co-occurring ataxia in one case, and pancerebellar syndrome complicated by action-induced dystonia in the other. We reviewed the literature on *TMEM240* variants linked to hyperkinetic disorders, comparing our cases to described phenotypes.

**Discussion:**

Adding to prior preliminary observations, our series highlights the relevance of hyperkinetic movements as clinically meaningful features of SCA21. *TMEM240* mutation should be included in the differential diagnosis of myoclonic dystonia and ataxia-dystonia syndromes.

## Introduction

Spinocerebellar ataxia 21 (SCA21) is a rare autosomal dominant neurodegenerative disorder caused by *de novo* occurrence or inherited transmission of heterozygous pathogenic variants in the Transmembrane Protein 240-encoding gene *TMEM240* [1]. The defining clinical features of the disease are progressive ataxia, akinesia, oculomotor disturbances, and dysarthria, often associated with concurrent cerebellar atrophy. The age of onset is variable, ranging from childhood to early adulthood, and so are the rate of progression and the accompanying extracerebellar manifestations, which can include cognitive impairment and psychiatric symptoms [2,3]. Hyperkinetic movement-disorder phenotypes in SCA21 have been increasingly rising to attention thanks to a series of recent contributions [2,3,4,5] which highlighted such presentations as more frequent than appreciated before. To date, two missense variants – p.Gly66Arg and p.Pro170Leu – have been the most consistently associated with hyperkinetic signs, including preliminary observations of myoclonus, action and postural tremor, and dystonia. In this work, we further outline the SCA21-related hyperkinetic movement-disorder spectrum by describing two unrelated probands and the mother of one of them, all carrying the recurrent pathogenic substitution p.Pro170Leu and sharing a heterogeneous presentation of dystonic features.

## Case descriptions

**Figure 1:**
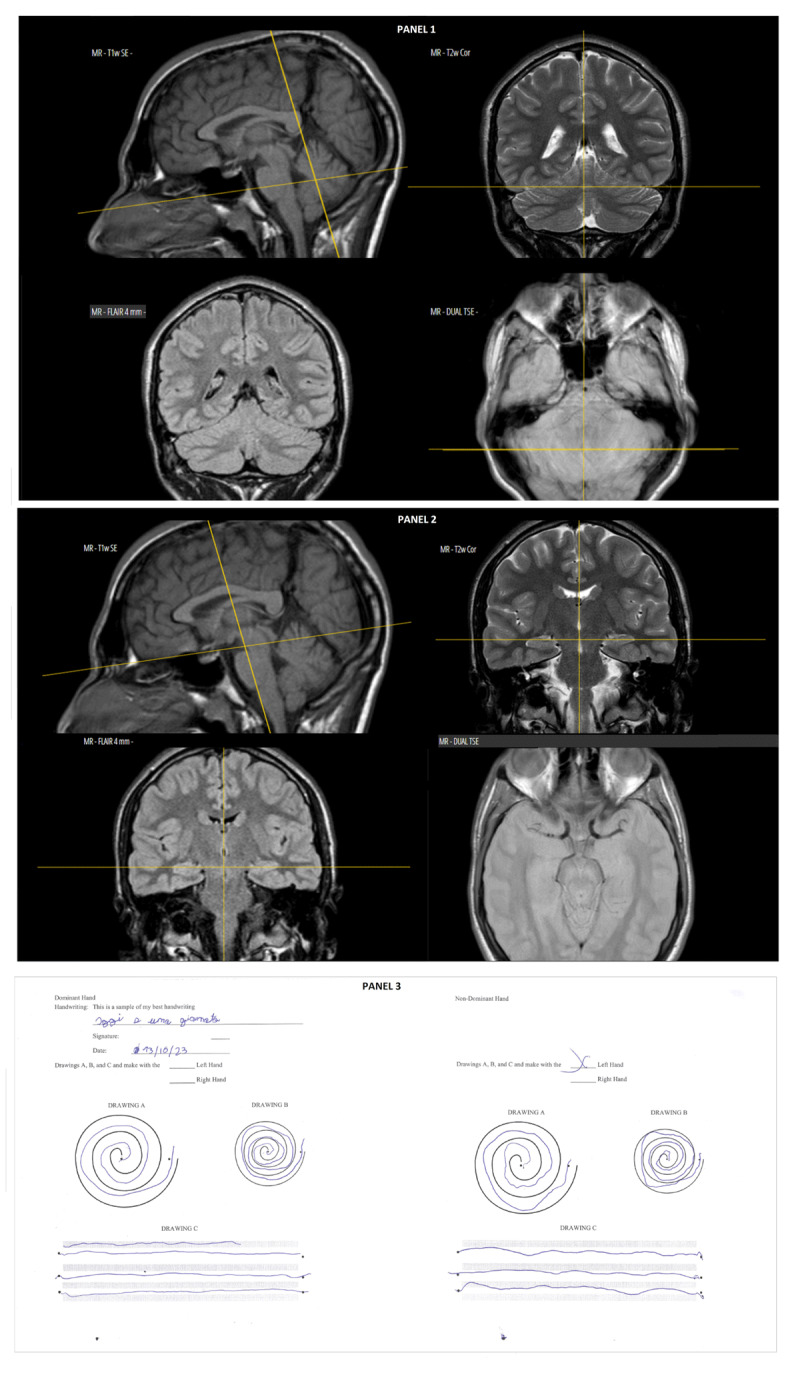
Normality of Brain MRI of first family’s proband, showing absence of cerebellar atrophy **(panel 1)** and absence of atrophy of the body of the hippocampus **(panel 2)**. A moderate task-specific tremor of writing and drawing is documented **(panel 3)**.

The first proband was a 31-year-old man. He had an unremarkable pregnancy and perinatal history; his psychomotor development was normal. From the age of 3 years, he developed a generalized myoclonus-dystonia disorder, which was initially most evident in the truncal and cervical regions, extending to the upper limbs, particularly while performing writing or drawing tasks with the right (dominant)-hand (Video 1, segment A, and B-limited to writing tasks). The patient attended school with a support teacher, and neuropsychological tests revealed impairments in executive functions (working memory, attention, concentration), inarticulate speech, dysgraphia, and dyslexia; during the late childhood, he developed impulsivity and anger management issues. His adjusted intellectual quotient (IQ), measured by the Form I of the Wechsler Adult Intelligence Scale (WAIS), scored 84 (at the lower limit of normal). He had severe difficulties in finding a suitable working role. Repeated neurological evaluations did not detect any ataxic or other cerebellar signs. Notably, his brain magnetic resonance imaging (MRI) was also normal, showing no signs of cerebellar, basal ganglia, hippocampus, or brainstem atrophy (Figure 1, Panels 1 and 2). The patient had no history of antipsychotic exposure. Before knowing the molecular diagnosis, gabapentin was selected as first line therapy and was administered in daily doses of 300 mg. Interestingly, the drug appeared to effectively and stably reduce the patient’s symptoms from generalized to mainly mild cervical-truncal myoclonic dystonia (Video 1, segment B; Figure 1, Panel 3). Specifically, a significant reduction in dystonic rapid movements with myoclonic features, involving the cranio-cervical and truncal regions and limbs, was observed a few months after the introduction of gabapentin. Because the patient felt comfortable with the symptom control achieved, to date no attempts have been made to modify his therapy with other drugs.

**Video 1:** Time-evolution of the neurological signs presented by the first proband. Two time periods are considered, referring to 2011, age 19 (segments A) (no neuropharmacological treatment), and to 2023, age 31, at the last available follow-up (segment B, under chronic treatment with Gabapentin 300 mg daily). Captions are embedded into the video. On note, the presence of diffuse dystonic rapid movements with myoclonic features with axial (cranio-cervical and truncal) and limbs distribution (segment A), showing a significant reduction under gabaergic treatment (segment B).

**Figure 2:**
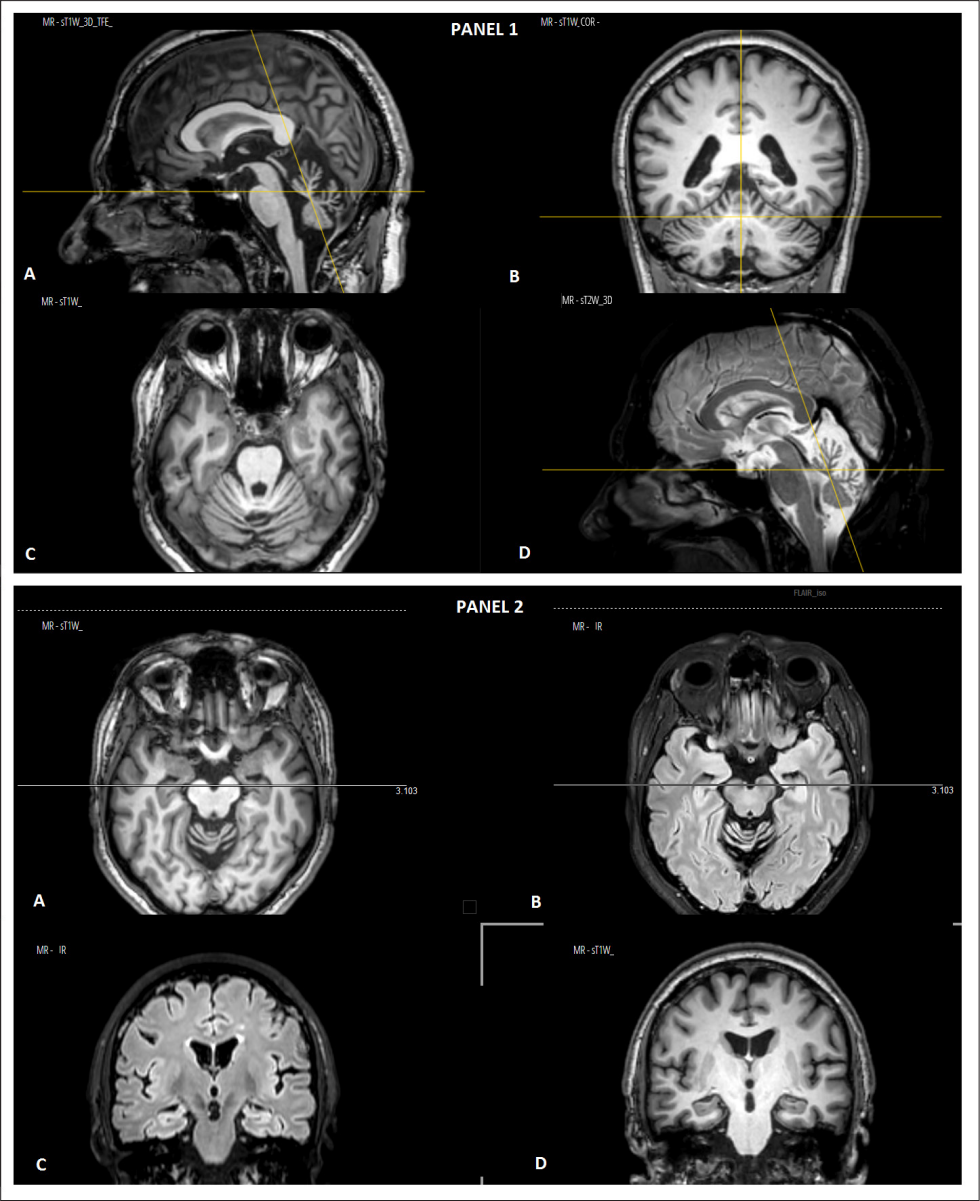
Panel 1. Brain MRI of the mother of the first family’s proband, showing prominent cerebellar atrophy, especially in the vermis. (A–C) T1-weighted images centered on the cerebellum on sagittal **(A)**, coronal **(B)** and axial **(C)** planes; **D)** sagittal T2-weighted image. Panel 2. MRI study showing moderate bilateral atrophy of the body of the hippocampus. (A, B) Axial images centered on the hippocampi on T1-weighted (A, D) and IR-w (B, C) images.

Family history of the first proband was positive for dystonia and ataxia in the maternal branch. His mother, who had no relevant medical history prior and whose development during childhood was reportedly normal, began manifesting neurological symptoms at 65 years of age, subjectively experiencing “internal tremors” in the lower limbs. Balance and gait impairment also appeared in the following months. Neurological examination at age 69 showed the presence of cerebellar signs (Video 2) with mild oculomotor disturbances, mild dysmetria and tremor in upper limbs and moderate dysmetria in lower limbs, and a broad-based gait with lower limbs action dystonia with myoclonic features (10/40 on the Scale for the Assessment and Rating of Ataxia – SARA). Notably, her speech was normal. Though she did not complain of any cognitive impairment, her adjusted Mini Mental State Examination score was 22.9/30. MRI showed the presence of cerebellar atrophy (Figure 2, Panel 1) and mild reduction in hippocampal volume (Figure 2, Panel 2). The patient also underwent a study of motor cortices excitability with paired-pulse magnetic stimulation, using conditioning stimuli to modify the response to the test stimulus. Normally, short intervals (1–5 ms) between the two stimuli cause a reduction in magnetic evoked potential, while longer intervals (10–30 ms) elicit a larger response compared to test stimulus alone. The patient showed reduced inhibition and an increased response to facilitatory stimuli, possibly related to GABA circuitry dysfunction. EMG was unremarkable.

**Video 2:** The neurological signs of Family 1 proband’s mother are shown (referring to 2023, age 69): this lady displays fragmented pursuits, with increased latency and mild slowing of saccades. Dysarthria is not present. As demonstrated by the patient reciting the Italian sentence “*Oggi è una bella giornata di sole*” (“Today is a beautiful day”) and the Italian numbers 1 to 10: “*uno, due, tre, quattro, cinque, sei, sette, otto, nove, dieci*”. Slight dysmetria and tremor in upper limbs, moderate dysmetria in lower limbs, and a mildly broad-based and slowed gait with some difficulties in turning but without support, are shown. On note, lower limbs action dystonia activated by the gait is shown: lower limbs dystonic features do not occur while patient standing or walking backward. The tightrope walking is difficult, mainly due to the activation of lower limbs action dystonia with myoclonic features. The feet-together stance is maintained with mild oscillations; Romberg sign is positive. Captions are embedded into the video.

The second proband was referred as a 68-year-old man with a long history of cerebellar disease and manifestation of limb dystonia. He was born at term after an uneventful pregnancy, with normal birth parameters. The acquisition of motor milestones was reportedly slightly delayed. From the age of 14, he began displaying signs of a slowly progressive pancerebellar syndrome, which at its full extent included severe standing and gait ataxia, asynergia, adiadochokinesia, ocular movements disturbance, and dysarthria in the form of scanning speech. Neurological assessment also revealed bilateral action-induced dystonia in the upper extremities and the left leg (Video 3). His most recent brain MRI documented severe cerebellar atrophy. His cognitive skills deteriorated over time as well, evolving into severe dementia at the later stages of the disease.

**Video 3:** Neurological assessment of the second proband. In the first segment, severe cerebellar dysarthria, cerebellar ataxia with adiadochokinesia and hypermetria on finger-to-finger test, and bilateral hand dystonia are observed. Oculomotor abnormalities including square wave jerks and oculomotor apraxia are evident. The second segment shows a very profound and unstable ataxic gait with a broad base and irregular steps. Dystonia of the left leg can also be seen.

Genomic sequencing analysis, performed according to established protocols and analysis pipelines [6], identified the *TMEM240* NM_001114748.2: c.509C>T, p.(Pro170Leu) heterozygous pathogenic variant in both probands. Variant segregation in the first family confirmed the suspected pattern of maternal inheritance. Written informed consent for publication of the clinical, molecular, and visual data was obtained from both probands and from the mother of the first proband, as appropriate and according to ethical guidelines.

## Discussion and conclusions

As a relatively recent addition to the group of spinocerebellar ataxias, SCA21 has been associated with hyperkinetic movement disorders for the first time in 2019 by Traschütz et al. [2], who described a small series of patients whose clinical presentations included myoclonic movements or action-induced tremor. Such observation was later confirmed by single cases reported by Riso et al. [3], Camargo et al. [4], and Cherian et al. [5], who extended the spectrum of the disorder to include myoclonic-dystonic manifestations. Although a general association between hyperkinetic disorders and the broad category of spinocerebellar ataxias has long been acknowledged [7,8], a correct estimate of the relevance of such relationship in the context of SCA21 is still limited, mainly due to the overall small number of described cases. To date, a total of 10 individuals from 6 unrelated SCA21 families have been reported showing hyperkinetic manifestations (Table 1). The two novel families described in this work represent a further, important validation of the phenotypic co-occurrence of ataxia and dystonia/myoclonic dystonia in SCA21, as they add consistency to the observations from previous reports, while also enhancing our understanding of both intrafamilial and interfamilial phenotypic variations in *TMEM240*-related disorders.

**Table 1:** Phenotypic comparison of reported SCA21 patients with hyperkinetic manifestations. CA: cerebellar atrophy; ID: intellectual disability; IQ: intelligence quotient.


	TRASCHÜTZ (2)	TRASCHÜTZ (2)	TRASCHÜTZ (2)	TRASCHÜTZ (2)	RISO (3)	CAMARGO (4)	CHERIAN (5)	THIS PAPER	THIS PAPER	THIS PAPER

**Patient**	A.II.2	A III.1	B II.1	C II.2	D II-5	Proband	Proband	Proband 1	Mother of Proband 1	Proband 2

**Mutation**	p.Pro170Leu	p.Pro170Leu	p.Pro170Leu	p.Gly66Arg	p.Gly66Arg	p.Pro170Leu	p.Gly66Arg	p.Pro170Leu	p.Pro170Leu	p.Pro170Leu

**Age at onset (years)**	40	2	32	3	0	51	3	3	65	4

**Age at last assessment (years)**	51	18	36	3	7	56	17	31	69	58

**Hyperkinetic phenotype**	Mini-myoclonus, mild myoclonus of arms/legs	Mini-myoclonus, mild myoclonus of arms/legs	Action tremor	Myoclonus	Distal dystonic posturing of the hands associated with jerky movements; intention and postural tremor	Symmetric tremor in the upper extremities and head, associated with subtle dystonic posture in the left hand	Right torticollis, bilateral upper limb dystonia, with superimposed spontaneous and action-induced upper limb myoclonus	Generalized myoclonus-dystonia	Lower limbs action dystonia with myoclonic features	Bilateral action-induced dystonia in the upper extremities and the left leg

**Typical cerebellar manifestations**	Gait and limb ataxia	Limb ataxia	Gait and limb ataxia	Gait and limb ataxia	Ataxia, dysmetria	Progressive ataxia, dysarthria, mild dysphagia	Absent	Absent	Progressive ataxia, mild oculomotor disturbances, mild dysmetria and tremor	Progressive pancerebellar syndrome

**Cognitive status**	Normal development, later decline	Normal	ID	ID	Global developmental delay	Normal	ID	IQ at the lower threshold of normality, required support teacher at school. Impairments in executive functions, language poverty, dysgraphia, and dyslexia.	Reportedly normal, but adjusted Mini Mental State Examination scored 22.9/30	Late onset cognitive deterioration, dementia

**Brain MRI**	CA	Normal	CA	Normal	CA, delayed myelination	Mild CA	Normal	Normal	CA and mild reduction of hippocampal volume	CA


Interestingly, the subset of SCA21-affected patients with hyperkinetic movements reported to date harbored either the p.Gly66Arg or the p.Pro170Leu recurrent pathogenic variants, raising the question whether non-ataxic movement disorders could be specific to these two variants. However, it should be noted that almost every other disease-causing variant identified thus far in *TMEM240* has been described only in single or single-family cases, making interpretations in this regard complicated. Nonetheless, the recognition of a growing number of cases caused by the same two variants represents a powerful opportunity to enhance our understanding of possible aetiopathological mechanisms and genotype-phenotype correlations of hyperkinetic movement disorders in SCA21.

Patients with the p.Gly66Arg substitution appear to show an earlier disease onset, while the age range of those carrying the p.Pro170Leu seems to be broader, spanning from childhood to even the sixth decade of life. Overall, hyperkinetic manifestations have been reported to occur predominantly in the upper limbs; however, a more generalized presentation, regardless of age, also appears to be possible, as demonstrated prominently by our first proband. The symptomatic burden is variable across patients, ranging from mild distal dystonia to generalized myoclonic dystonia. In this regard, allelic heterogeneity does not appear to be the primary factor, as the differences, both interfamilial and intrafamilial, among individuals with the same variant can be more pronounced than those between patients carrying different variants, especially when considering the overall SCA21 phenotype.

The first family of our series is a major example of such interfamilial variability, as the proband exhibited generalized myoclonic dystonia with no sign of cerebellar involvement or organic degeneration, while his mother presented only mild, action-induced lower limbs myoclonic dystonia, in the context of a more typical SCA21 progression. A difference can be seen also with respect to the other proband of our series, who, despite carrying the same p.Pro170Leu variant, displayed the full extent of both the hyperkinetic and the more typical cerebellar components of the disorder. In parallel, the very early-onset, generalized myoclonic dystonia phenotype developed by the first proband is reminiscent of those described by Riso and Cherian, who both carried the p.Gly66Arg variant. His clinical history, corroborated by normal neuroimaging findings, also supports the hypothesis, formulated by Cherian et al. in their report, that ataxia may be an unexpectedly inconsistent feature in SCA21 patients, greatly expanding the scope of differential diagnosis for this disorder. Conversely, the data at our disposal do not fully support another conclusion by the same authors regarding cognitive impairment, which they claimed to be a mandatory feature in SCA21 patients with myoclonic dystonia: although the two oldest among our participants did eventually develop different degrees of cognitive decline, for most of their lifespan their psychomotor development and cognitive skills were considered normal or at the lower limits of normality. Unimpaired cognitive capabilities in SCA21 patients had been previously reported by Traschütz and Camargo.

Although the actual function of the TMEM240 transmembrane protein is yet to be completely understood, a possible involvement in modulating ion channel function of neuronal synaptic membranes has been postulated, similarly to what observed in other SCAs caused by gain-of-function point mutations in channel-encoding genes [9]. In this regard, a direct or indirect influence on GABAergic transmission, which has been suggested to be reduced in SCA1 and is known to be involved in other genetic movement disorders primarily characterized myoclonic-dystonic manifestations [10], could represent the connecting factor between *TMEM240* variants and hyperkinetic movement disorders in SCA21 patients. The good response to gabapentin showed by our first proband represents a supporting, although anecdotal, evidence to such hypothesis, and could encourage a new line of investigation on both the clinical and basic research sides.

In conclusion, the two families described in this paper contribute to the existing evidence that hyperkinetic movement disorders, especially dystonia and myoclonus, should be considered a relevant feature within the phenotypic spectrum of SCA21, particularly in relation to the recurrent p.Pro170Leu variant. They also exemplify the substantial variability, both intrafamilial and interfamilial, of the phenotypes associated with this condition. Such expanding clinical spectrum also broadens the range of differential diagnosis of SCA21, highlighting the importance of unbiased molecular approaches when investigating patients with a suspicion of either neurodegenerative disorder or movement disorder.

## Data Accessibility Statement

The data reported in this study are available upon reasonable request to the corresponding author.
